# Case Report: Optic nerve infiltration associated with sequential CRVO and CRAO in isolated CNS relapse of AML

**DOI:** 10.3389/fmed.2026.1853833

**Published:** 2026-06-12

**Authors:** Yu Wang, Yiwei Li, Jiaxuan Zhang, Bojia Niu, Yujie Jiang, Ying Xiao

**Affiliations:** 1 Department of Ophthalmology, Shandong Provincial Hospital Affiliated to Shandong First Medical University, Jinan, Shandong, China; 2 Department of Hematology, Shandong Provincial Hospital Affiliated to Shandong First Medical University, Jinan, Shandong, China

**Keywords:** acute myeloid leukemia, case report, central nervous system relapse, central retinal artery occlusion, central retinal vein occlusion, optic nerve infiltration

## Abstract

Optic nerve infiltration as the initial manifestation of isolated central nervous system (CNS) relapse in acute myeloid leukemia (AML) has rarely been reported. We present the case of a 70-years-old man with AML in clinical remission who presented with the sudden onset of a visual field defect and vision loss in the left eye. Initially the patient was diagnosed with non-arteritic anterior ischemic optic neuropathy (NAION) at a local hospital based on findings of decreased vision, optic disc edema and an inferior sectoral visual field defect connected to the physiological blind spot in the left eye, the patient subsequently progressed rapidly to no light perception. Fundus examination showed sequential features consistent with central retinal vein occlusion (CRVO) followed by central retinal artery occlusion (CRAO). Magnetic resonance imaging (MRI) demonstrated thickening and enhancement of the left optic nerve with involvement of the intraconal fat. Cerebrospinal fluid (CSF) cytology confirmed leukemic infiltration, while bone marrow aspiration remained negative for malignant cells, establishing a definitive diagnosis of isolated CNS relapse with optic nerve infiltration. Following intrathecal and systemic chemotherapy, leukemic cells were cleared from the CSF, although the left eye remained without light perception. This case describes the previously unreported sequential onset of optic nerve infiltration, CRVO, and subsequent CRAO in AML, highlighting the need for clinicians to recognize such rare ocular manifestations to detect occult leukemia recurrence early. Atypical or unexplained optic neuropathy in AML patients should raise suspicion of leukemic infiltration. Timely performance of CSF cytology and cranial MRI, together with a multidisciplinary diagnostic and therapeutic approach, is essential for improving visual prognosis and survival rate.

## Introduction

Leukemias are heterogeneous hematopoietic stem cell malignancies characterized by aberrant bone marrow blood cell proliferation. Acute myeloid leukemia (AML) is a clonal hematopoietic disease marked by uncontrolled expansion of immature myeloid cells. Leukemic cells can infiltrate multiple extramedullary organs through hematogenous spread and direct infiltration (1).

Central nervous system (CNS) leukemia is a common form of extramedullary relapse in leukemia. CNS leukemia is formally defined as the presence of leukemic cells (blasts) in the cerebrospinal fluid (CSF) and/or the identification of leukemic infiltrates within the brain parenchyma, cranial nerves, or spinal cord. Ocular involvement, specifically infiltration of the optic nerve, retina, or other intraocular structures, is considered a form of CNS leukemia. This results from the optic nerve being an extension of the central nervous system, and the eye is connected via the optic canal, allowing leukemic cells to spread directly from the CSF or via hematogenous routes to these sites (2, 3). Its presence signifies that the leukemic cells have breached the blood-brain or blood-retinal barrier, classifying it as CNS involvement.

In cases of ocular involvement, fundus abnormalities are the most commonly documented symptoms of leukemia, whereas optic nerve infiltration is exceptionally rare. It may occur at any stage of the disease and can even serve as the only manifestation of extramedullary relapse, sometimes preceding hematological relapse by several months. Its presence is associated with a poorer prognosis, correlating with higher rates of bone marrow relapse and CNS relapse (4, 5).

Despite advances in current therapies for AML, CNS involvement remains a significant clinical challenge and is associated with severe complications and increased mortality. Herein, we report a rare case of CNS extramedullary relapse of AML in which optic nerve edema and retinopathy were the initial manifestations. The patient was initially diagnosed with ischemic optic neuropathy, resulting in delayed treatment and permanent vision loss. This case highlights the critical importance of CSF analysis in the neurological evaluation of patients with a known history of AML. For AML patients exhibiting visual symptoms, ophthalmologists should promptly consider the possibility of CNS extramedullary relapse and initiate timely multidisciplinary management in collaboration with hematologists to optimize visual and clinical outcomes.

## Case description

**FIGURE 1:**
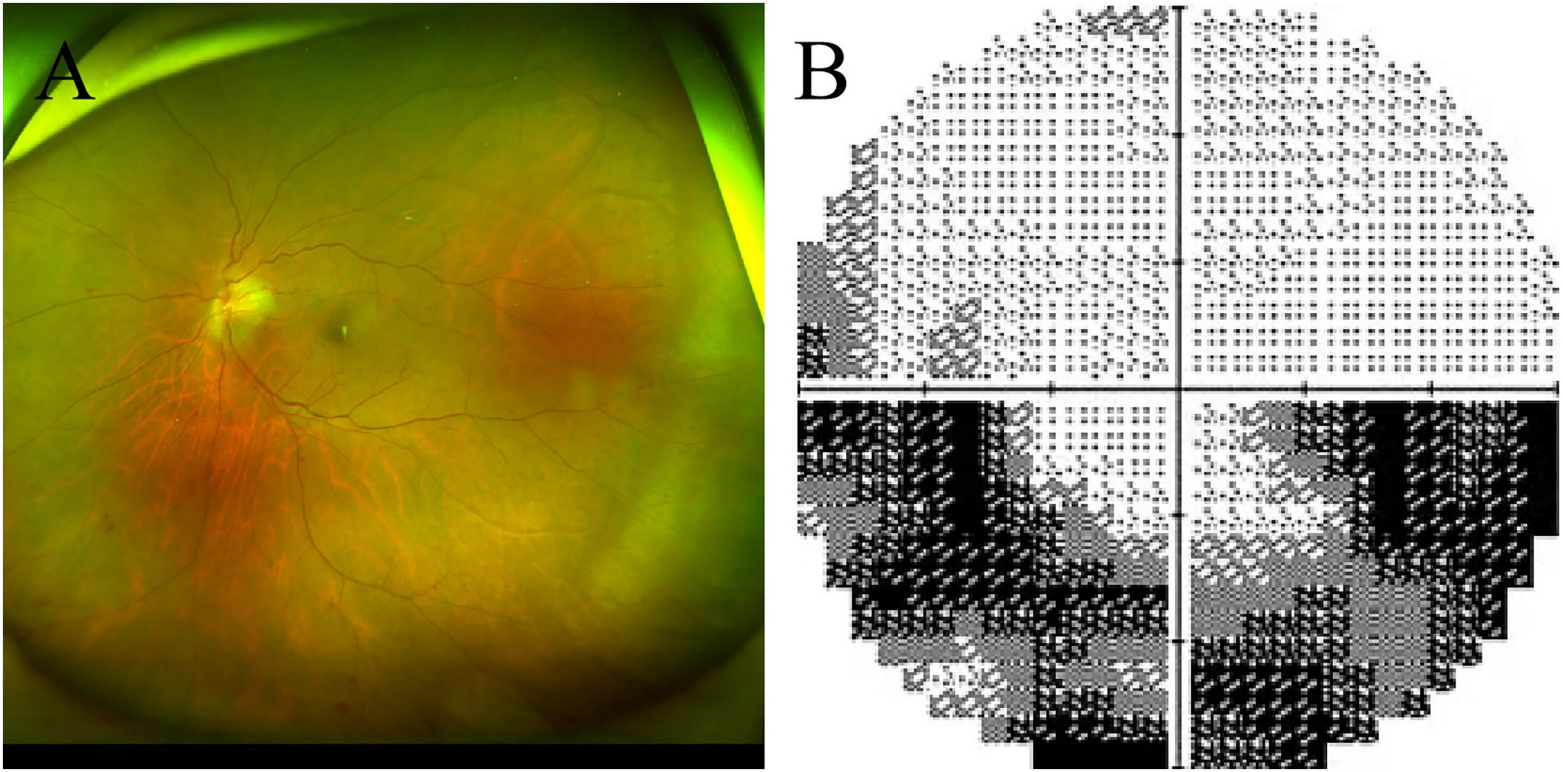
Initial presentation (local hospital, left eye): Mildly pale and swollen optic disc **(A)**; visual field testing revealed an inferior visual field defect in the left eye **(B)**. **(A)** Obtained from a pseudo-color Optos ultra-widefield image.

A 70-years-old man presented with sudden reduced vision accompanied by a sense of inferior scotoma in his left eye for more than 10 days. Recorded visual acuity in the left eye was 20/80 at that time. He was initially diagnosed with non-arteritic anterior ischemic optic neuropathy (NAION) at local hospital, possibly based on fundus findings of a mildly pale and swollen optic disc (Figure 1A) along with an inferior visual field defect (Figure 1B), as well as a history of long-standing diabetes. Treatment at the local hospital included retrobulbar injections of raceanisodamine hydrochloride and dexamethasone. Due to persistent symptoms, orbital pain, and rapid progression of vision loss, he was referred to our department for further evaluation and management.

**FIGURE 2:**
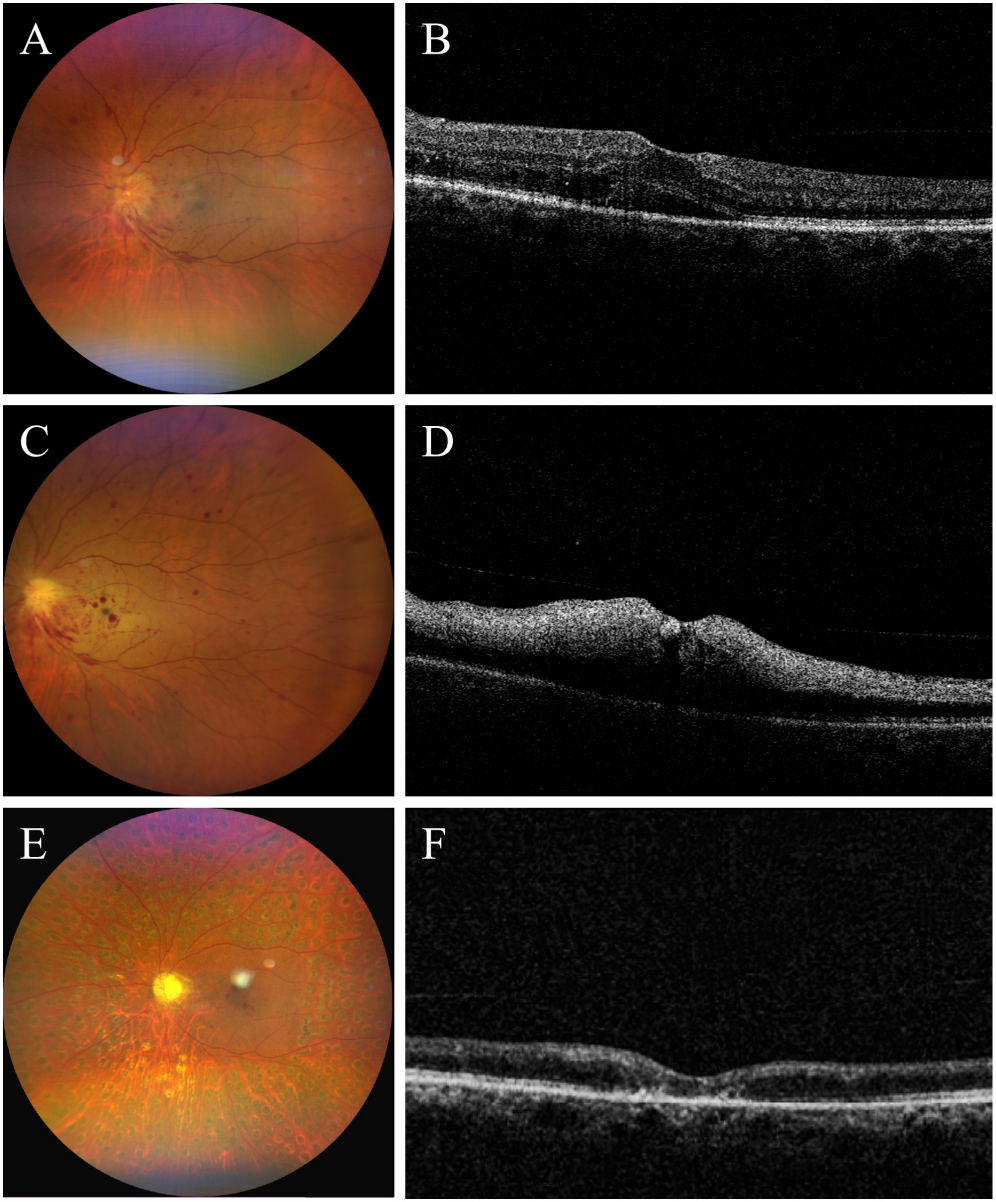
Initial presentation (left eye): Markedly swollen and pale optic disc with linear hemorrhages; tortuous dilated veins and extensive intraretinal hemorrhages (flame, punctate, patchy) in all four quadrants **(A)**; OCT revealed mild macular edema and subretinal fluid beneath the fovea **(B)**; 1-week follow-up (left eye): Aggravated papilledema, increased retinal hemorrhages, and new macular cherry-red spot **(C)**; OCT revealed diffuse hyperreflectivity of the inner retinal layers, consistent with the findings of CRAO **(D)**; 3-months follow-up (left eye): Attenuated papilledema with disc pallor; residual linear hemorrhages; resolved cherry-red spot; increased patchy macular hemorrhages **(E)**; OCT revealed retinal thinning and atrophy **(F)**. **(A,C,E)** Are true-color ultra-widefield fundus images acquired with the CLARUS 500 system.

Upon admission to our hospital, the patient’s left eye had progressed to no light perception accompanied by a positive relative afferent pupillary defect. Further investigation of the patient’s medical history revealed a diagnosis of AML with CBFβ/MYH11 fusion gene and KIT D816V mutation 8 months prior, at which time his white blood cell count was 61.9 × 10^9^/L. He had completed six cycles of chemotherapy and was in clinical remission at presentation. CSF examination before the first consolidation therapy showed no abnormalities. Given the KIT D816V mutation and insufficient reduction of CBFβ::MYH11 expression, hematologists recommended an avapritinib-based regimen and allogeneic hematopoietic stem cell transplantation (Allo-HSCT), which was declined by the patient’s family due to financial burden. Considering the patient’s poor general condition and intolerance to HD-AraC therapy, the VA regimen was ultimately selected. His medical history included diabetes mellitus of over 10 years’ duration, controlled with acarbose and metformin. The anterior segment examination was unremarkable in both eyes. Fundus examination of the right eye revealed no significant abnormalities. In the left eye, however, the optic disc appeared markedly swollen and pale, with linear hemorrhages along its margin. All four retinal quadrants exhibited retinal venous tortuosity and dilatation, together with a noticeable increase in flame-shaped, punctate and patchy intraretinal hemorrhages compared to 1 week before (Figure 2A). Optical coherence tomography (OCT) revealed mild macular edema and subretinal fluid beneath the fovea (Figure 2B). Additionally, the patient remained afebrile with normal inflammatory markers, including ESR and CRP.

Given these findings, a high level of clinical suspicion was raised for leukemic infiltration of the left optic nerve alongside concurrent central retinal vein occlusion (CRVO). Urgent magnetic resonance imaging (MRI) of the brain and optic nerves, along with CSF cytological examination, was therefore arranged.

**FIGURE 3:**
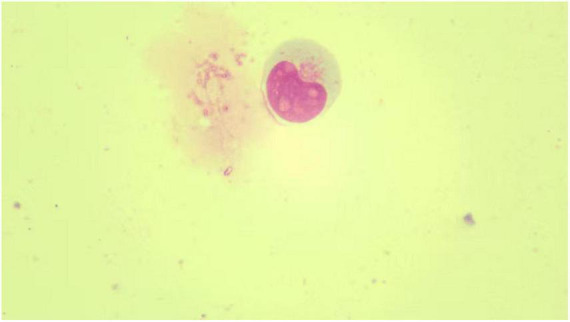
Cytospinned sediment of cerebrospinal fluid shows a large blast cell with high nuclear-to-cytoplasmic ratio, irregular nuclear morphology, and prominent nucleoli (MGG, 40× objective, original magnification ×400).

MRI of the brain and optic nerve revealed thickening of the left intraorbital optic nerve, accompanied by enhancement of the optic nerve sheath (Supplementary Figure 1). Leukemic cells were detected in CSF cytology (Figure 3), and flow cytometry of cerebrospinal fluid also revealed abnormal myeloid blasts. Furthermore, the CSF protein level was confirmed to be elevated, with a measured value of 0.57 g/L (reference range: 0.15–0.45 g/L). All these supported the diagnosis of CNS relapse, classified as an extramedullary relapse originating from the optic nerve. The patient subsequently received systemic chemotherapy with the venetoclax combined azacitidine (VA) regimen and intrathecal triple chemotherapy every 3 days, comprising methotrexate, cytarabine, and dexamethasone. However, as orbital MRI revealed no discernible tumor mass, target volume delineation for radiotherapy could not be performed, and irradiation of the left eye was therefore deferred.

One week later, physical examination revealed aggravated optic disc edema, increased retinal hemorrhages, and the emergence of a macular cherry-red spot in the patient’s left eye (Figure 2C), and OCT revealed diffuse hyperreflectivity of the inner retinal layers, indicative of the onset of central retinal artery occlusion (CRAO) (Figure 2D). Fundus fluorescein angiography (FFA) demonstrated a serious delay in arterial filling (up to 8 min), extensive retinal non-perfusion, along with leakage from the optic disc (Supplementary Figure 2). In view of the substantial ischemic burden and high risk of neovascular complications, panretinal photocoagulation (PRP) was promptly performed to reduce the likelihood of neovascular glaucoma, vitreous hemorrhage, and other sequelae. After three sessions of intrathecal chemotherapy, CSF cytology and flow cytometry became negative. The CSF protein content decreased compared to before, but remained higher than the upper limit of normal. During the subsequent treatment, this patient opted for a regimen combining teniposide and cytarabine, which are known to have relatively good penetration across the blood-brain barrier. Concurrently, the patient continued to undergo lumbar punctures with intrathecal chemotherapy for a total of six consecutive sessions. Follow-up CSF examinations all returned normal results.

During the 3-months follow-up, the right eye remained essentially unremarkable. In contrast, examination of the left eye indicated attenuation of optic disc edema with the emergence of optic disc pallor. The majority of the disc margin hemorrhages had resolved, the cherry-red spot had diminished, and patchy macular hemorrhages had increased compared with the previous examination (Figure 2E). OCT revealed retinal thinning and atrophy (Figure 2F). At the final follow-up, partial resorption of the patchy macular hemorrhages in the left eye was noted, with the remaining findings largely unchanged from the previous examination. The patient’s visual acuity, however, has remained unchanged to date.

## Discussion

Acute myeloid leukemia is a malignant clonal hematopoietic disorder characterized by unregulated proliferation and impaired differentiation of immature myeloid cells, resulting in ineffective hematopoiesis, life-threatening cytopenia, and transfusion dependence (6). Extramedullary relapse in AML is uncommon yet clinically significant. The most frequently affected sites are the skin and soft tissues, followed by the leptomeninges, testes, and ocular structures (7).

Previous studies have shown that ocular involvement occurs in approximately 48.4%–68% of AML patients, in contrast to 30.8%–42% in acute lymphoblastic leukemia (ALL), 10%–33% in chronic lymphocytic leukemia (CLL), and 13%–25% in chronic myeloid leukemia (CML) (8–11). Ocular involvement may appear after the systemic diagnosis of leukemia, constitute its initial presentation, or emerge as the first sign of relapse after remission. The underlying mechanisms include direct leukemic cell infiltration and/or hematogenous spread (2, 3, 12–14). Among all manifestations, leukemic retinopathy represents the most prevalent ocular finding. First described by Liebreich in the 1860s, it remains one of the most frequently observed ocular manifestations of leukemia (12). In AML patients exhibiting ocular involvement, vitreous hemorrhage (21%) and subretinal hemorrhage (8%) are among the most frequently reported manifestations (8).

Previous reports indicated that leukemic optic neuropathy is a relatively rare but clinically significant finding, often serving as an indicator of CNS involvement. In leukemia patients, primary optic nerve involvement typically presents as optic disc edema and has been reported in approximately 13%–18% of cases (5). According to a histopathological study by Allen and Straatsma, optic nerve involvement was observed in about 34% of ocular leukemic cases, predominantly in acute leukemias (15). ALL is the subtype most frequently associated with optic nerve involvement, whereas such manifestations are relatively uncommon in AML, possibly due to the higher incidence of CNS involvement in ALL. Studies indicate that approximately 5%–15% of adult ALL patients present with CNS involvement at diagnosis, and about 5% experience isolated CNS relapse after achieving remission. In contrast, CNS involvement in adult AML occurs in only about 1.7%–5.06% of patients throughout the disease course (16).

Although rare, optic nerve infiltration in AML has been documented in a few case reports. For instance, Patel et al. described a 48-years-old man with relapsed AML and MLL gene rearrangement, who presented with acute unilateral vision loss as the initial manifestation. CSF cytology detected numerous leukemic blasts. Despite intrathecal chemotherapy and whole-brain radiotherapy, the patient progressed to bilateral blindness and ultimately died from disease progression (7). In contrast, Quann et al. reported a 35-years-old woman with AML who developed leukemic optic nerve infiltration after hematopoietic stem cell transplantation, manifesting as sudden vision loss. Subsequent to local radiotherapy and intrathecal chemotherapy, she achieved cytological remission with partial visual recovery (17). These cases underscore the considerable heterogeneity in the clinical course and visual outcomes of leukemic optic neuropathy in AML.

Multiple independent risk factors for CNS leukemia in AML have been identified, including younger age, elevated white blood cell count and lactate dehydrogenase (LDH) levels at diagnosis, chromosomal abnormalities involving 11q23, and FLT3-ITD mutations (18–20). Furthermore, patients with myelomonocytic (M4) or monocytic (M5) AML are more susceptible to CNS involvement (21). The present case involved a patient with AML-M4 and marked leukocytosis (WBC: 61.9 × 10^9^/L) during his first diagnosis of AML, both of which may have contributed to the risk of CNS invasion. Unilateral optic nerve infiltration with sudden visual loss is very rare, as it was in this case report.

The initial NAION diagnosis at the local hospital was clinically plausible. It was primarily based on the typical presentation, such as unilateral involvement, a pale edematous optic disc and characteristic visual field defects, coupled with the patient’s profile as an older male with long-standing diabetes, all of which represented established risk factors for NAION. Diabetes induced systemic small-vessel sclerosis and luminal stenosis, together with AML-related hypercoagulability, were all speculated for the hypoperfusion of the short posterior ciliary arteries and subsequent NAION (22–24). However, the complete loss of light perception within days was inconsistent with typical NAION, strongly indicating the presence of other underlying pathology. Consequently, differential diagnosis was pursued, including giant cell arteritis associated AION and intracranial hypertension related papilledema. AION was excluded by patient’s afebrile status, normal inflammatory markers (ESR/CRP), and the lack of arteritic changes on carotid ultrasonography. Similarly, normal lumbar puncture pressures and unremarkable cranial MRI effectively ruled out intracranial hypertension, which typically presents with bilateral disc swelling. Ultimately, the rapid unilateral vision loss, which was refractory to conventional therapy, raised suspicion for leukemic optic nerve infiltration for this patient.

Leukemic optic nerve infiltration was classified into prelaminar and retrolaminar previously (12). Prelaminar optic nerve infiltration generally manifests as superficial, fluffy infiltrates on the lamina cribrosa, with relatively preserved visual acuity unless the macula is involved. In contrast, retrolaminar involvement typically presents with marked optic disc edema, which frequently correlate with severe visual impairment (5, 12). We hypothesized that prelaminar optic nerve infiltration had already presented at the initial visit to the local hospital. In the absence of prompt systemic chemotherapy, this process likely progressed aggressively to the retrolaminar stage within days, which had been proved by CSF cytology.

This case documents a unique sequential onset of optic nerve infiltration, CRVO and subsequent CRAO in AML, which, to our knowledge, has not been reported previously. This is likely an event of combined retinal vascular occlusion, which may result from complex infiltration-related pathological mechanisms. Retinal vascular occlusion has been previously documented in association with leukemic optic neuropathy. Lin et al. described a pediatric patient who developed bilateral CRAO within hours after being diagnosed with leukemic optic neuropathy (25). Similarly, a 7-years-old patient with B-ALL in remission presented with sudden vision loss accompanied by optic disc edema, CRAO and CRVO (26). Zhao et al. reported a rare case of AML in which unilateral optic nerve infiltration combined with CRAO served as the initial manifestation of CNS relapse after hematopoietic stem cell transplantation (27), eventually resulting in retinal atrophy and permanent vision loss.

In the present case, we postulated the combined vascular occlusion can arise from multiple etiologies, including direct leukemic infiltration, as well as secondary alterations driven by hypercoagulability, leukostasis and abnormal hemorheology. First, leukemia cells within the optic canal likely invaded retinal veins, inducing damage of venous wall and obstruction of CSF outflow (26, 28). AML-associated hyperviscosity and leukostasis resulting from markedly elevated circulating leukocytes or platelets (8, 12), diabetes-related hyperglycemia and endothelial injury, could also be attributed to hypoperfusion and vascular stasis, further leading to capillary non-perfusion, microvascular remodeling and vascular occlusion (29, 30). These synergistic mechanisms might account for the clinical manifestations of CRVO, such as venous engorgement and retinal hemorrhage, as well as worsening disc edema. As nerve fiber destruction and venous occlusion progressed, elevated pressure within the optic nerve sheath likely compromised central retinal artery perfusion. This mechanism, among above factors, may have precipitated the central retinal artery occlusion. Alternatively, CRAO might also result from direct embolization by neoplastic cells (28).

In another report, Khair et al. described a patient with relapsed AML who presented with bilateral retinal arterial occlusion as the first sign of CNS involvement, in the absence of obvious optic disc edema or retinal infiltration (31), which suggest that retinal vascular occlusion may arise from direct leukemic infiltration of the microvasculature. Such vascular occlusions may occur with or without optic disc edema or infiltration, and may even precede clinically detectable CNS relapse. In this case, rapid visual deterioration may not be caused by optic nerve infiltration alone; rather, retinal arterial occlusion likely plays a predominant role. For our patient, infiltrative optic neuropathy, which occurred before CRAO, was considered the primary cause of acute vision loss, rather than the retinal arterial occlusion itself.

Compared with ALL, CNS involvement is considerably less frequent in AML. CNS relapse can occur prior to clinically evident bone marrow recurrence. Given these considerations, the presence of optic disc edema or retinal vascular occlusion in an AML patient should raise immediate suspicion for CNS involvement and warrants a comprehensive neurological evaluation, given its significant prognostic and therapeutic implications (4, 5).

The optic nerve, similar to other intraocular structures, may serve as a pharmacologic “sanctuary site” for leukemic cells due to the limited penetration of systemic chemotherapeutic agents into this region (32). Compared with systemic chemotherapy alone, local radiotherapy may reduce tumor burden within the narrow optic canal and thereby improve the accessibility and efficacy of chemotherapeutic agents against residual leukemic cells. However, in this case, orbital MRI did not demonstrate a well-defined mass, making accurate delineation of the radiotherapy target volume difficult. Consequently, orbital radiotherapy was not performed. Following three courses of intrathecal triple chemotherapy, leukemic cells were cleared from the CSF, although no recovery of visual acuity was observed.

Previous studies suggest that early intervention may influence visual outcomes. Albert Hu et al. reported an adult patient with B-ALL who underwent orbital stereotactic radiotherapy immediately after leukemic optic nerve infiltration was confirmed on MRI, leading to complete visual recovery within 2 weeks and without vascular occlusive complications (33). In contrast, the AML patient reported by Patel et al. experienced rapid visual deterioration and ultimately died from disease progression (7). This unfavorable outcome was attributed to high-risk cytogenetic features and the presence of CRAO-a finding also present in our case. These observations suggest that the occurrence of CRAO may indicate irreversible visual damage and poor prognosis. The favorable outcome reported by Quann et al. may be related to prior allogeneic hematopoietic stem cell transplantation and stable donor-derived hematopoiesis (17). In our case, prolonged leukemic infiltration of the optic nerve and occlusion of retinal vascular, coupled with the absence of local radiotherapy, ultimately resulted in macular atrophy, making visual recovery highly unlikely.

In conclusion, optic nerve infiltration can serve as the early clinical manifestation of relapse in AML patients in remission. When visual symptoms develop in AML patients, clinicians should maintain a high index of suspicion for leukemic involvement and promptly arrange CSF cytological examination and cranial MRI to confirm the diagnosis. Early recognition is crucial not only to prevent irreversible vision loss but also to improve overall survival. Close collaboration between ophthalmologists and hematologists is critical for the early identification of ocular involvement and optimal disease management. To our knowledge, the sequential progression from optic nerve infiltration to CRVO and then to CRAO has not been previously reported in AML. Further studies involving larger cohorts are warranted to clarify the prognostic implications of ocular manifestations in this population.

## Data Availability

The original contributions presented in this study are included in this article/Supplementary material, further inquiries can be directed to the corresponding authors.
