# Supplementary material for: Case Report: Optic nerve infiltration associated with sequential CRVO and CRAO in isolated CNS relapse of AML
Source: Front Med (Lausanne). 2026 Jun 12;13:1853833. doi: 10.3389/fmed.2026.1853833 (PMC13303145; doi:10.3389/fmed.2026.1853833)
Supplement: Supplementary file 1 [file Table_1.docx]

# Supplemental Materials


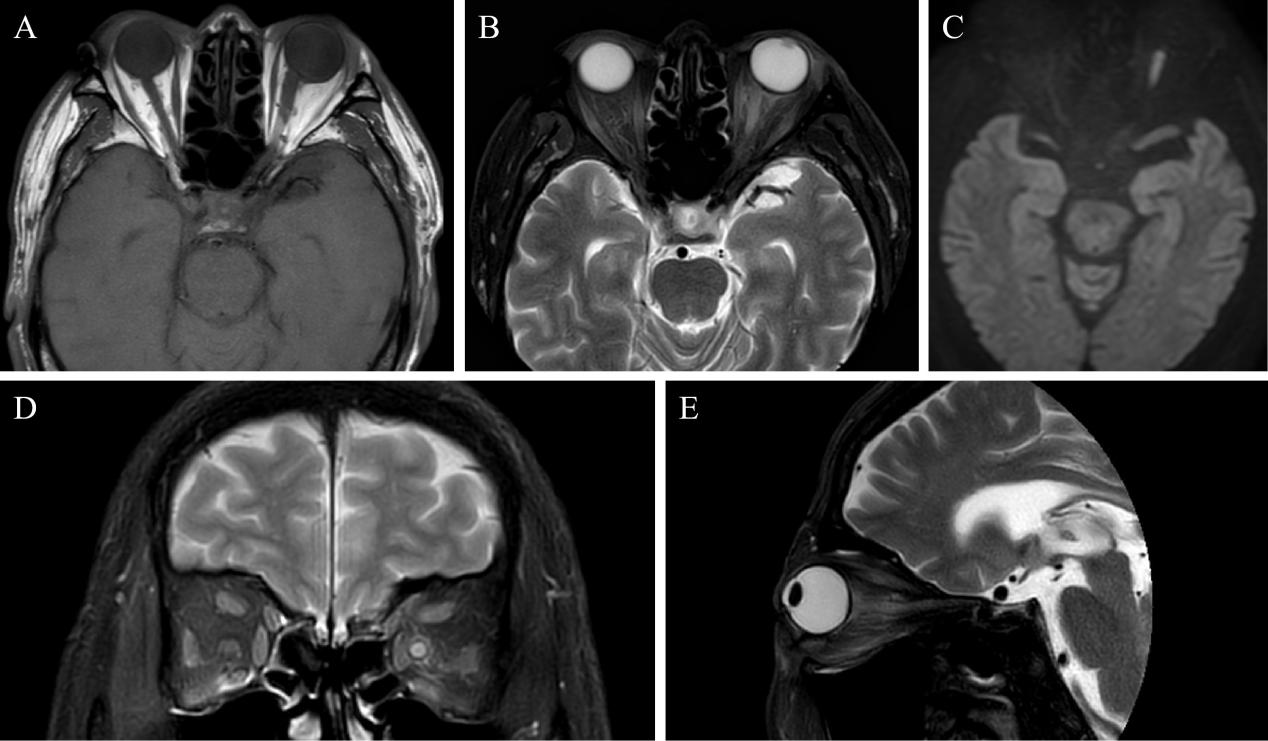


Supplementary figure 1: Axial pre-contrast T1-weighted (A) and T2-weighted (B) orbital MRI demonstrates diffuse thickening of the left intraorbital optic nerve, which is hypointense on T1 and hyperintense on T2, with associated optic nerve sheath distension and intraconal fat space infiltration. Coronal (D) and left orbital sagittal (E) T2-weighted images further delineate the longitudinal range of signal abnormality, whereas the corresponding axial diffusion-weighted imaging (C) demonstrates restricted diffusion within the involved optic nerve.


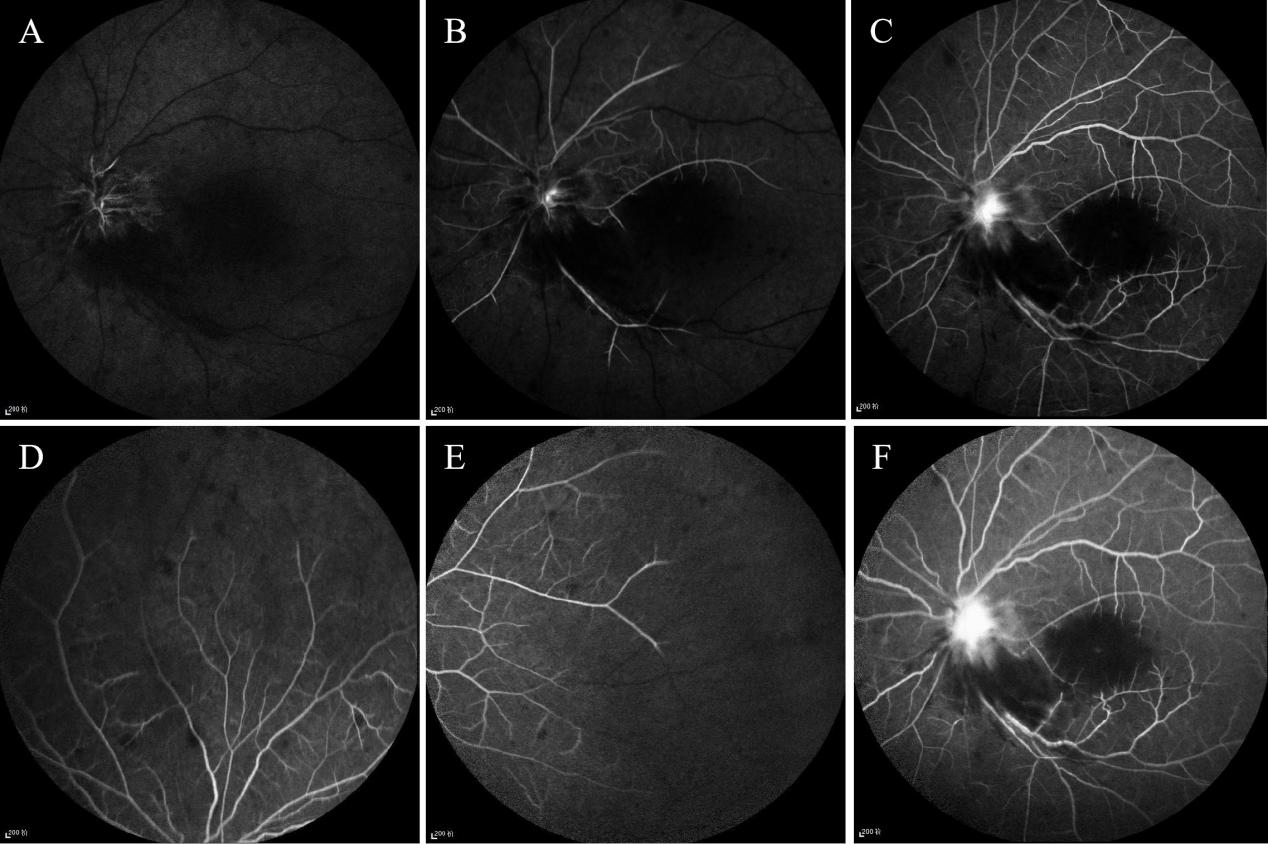


Supplementary figure 2: Sequential fluorescein angiography (FA) findings. At 30 s, only optic disc vessels were perfused, with hypofluorescence of the posterior pole (A). At 1 min 29 s, most posterior pole vessels remained unperfused. The superotemporal vein exhibits laminar flow, while hemorrhage beneath the optic disc caused fluorescence blockage. Note the caliber irregularity of the inferotemporal artery (B). At 8 min 8 s, persistent partial non-perfusion and disc leakage were noted in the posterior pole (C). At 8 min 21 s (D) and 9 min 56 s (E), abnormal termination of peripheral vascular and extensive non-perfusion areas was observed. At 19 min, optic disc showed marked leakage and hyperfluorescence; the posterior pole remained hypofluorescent, and inferotemporal vein exhibited tortuous dilatation with wall staining (F).
